# Giant Right Coronary Artery Aneurysm Incidentally Found During Stroke Evaluation Through Echocardiography

**DOI:** 10.7759/cureus.51656

**Published:** 2024-01-04

**Authors:** Jessica M Gonzalez, Gabriel Lowenhaar, Prasad Chalasani

**Affiliations:** 1 Internal Medicine, Brown University, Providence, USA; 2 Emergency Medicine, Brown University, Providence, USA; 3 Cardiology, Florida State University College of Medicine, Fort Pierce, USA

**Keywords:** giant coronary artery aneursym, transthoracic echocardiogram, coronary aneurysm discovered poststroke, echocardiogram detecting coronary aneurysm, coronary artery aneurysm

## Abstract

Giant coronary artery aneurysms (CAAs) are rare forms of coronary artery disease. An 82-year-old man presented to the hospital with generalized weakness, arm numbness, and dizziness and was found to have a multi-infarct stroke. A transthoracic echocardiogram was obtained to determine a possible cardiovascular etiology of his stroke. However, it did not reveal thrombi or vegetation; instead, it showed a ring-like structure adjacent to the tricuspid valve that appeared to be a large right atrial cyst. A transesophageal echocardiogram was performed localizing the ring-like mass near the tricuspid annulus. Cardiac catheterization revealed aneurysms of the coronary arteries with complete distal occlusion of the left anterior descending artery (LAD), an aneurysmal left circumflex, and a right coronary artery with a very large aneurysm without signs of thrombus or flow-limiting lesion. CAAs are usually found through cardiac catheterization. Echocardiography may be a novel way of identifying CAAs.

## Introduction

Coronary artery aneurysms (CAAs) are dilations of arteries that exceed the diameter of other adjacent arteries by 1.5 times or are 50% greater in arterial diameter compared with an adjacent arterial segment [1]. Giant CAAs have a vessel dilation of 2 cm or greater in diameter [2]. The incidence of CAAs is between 0.3-5%. Giant CAAs are exceedingly rare with a reported incidence in a cardiac surgical population of 0.02% [1]. CAAs appear to affect the right coronary artery (40%) most commonly, secondly, the left anterior descending artery (32%), and rarely, the left main coronary artery (3.5%) [2]. Factors that can lead to their development include congenital disorders, atherosclerosis, genetic causes, overexpression of enzymes such as ACE, autoimmune/inflammatory changes, connective tissue disorders, dynamic wall stress changes, Takayasu arteritis, Kawasaki disease, and percutaneous coronary intervention [2-3]. Although most CAAs are silent, various clinical presentations have been reported in previous literature. Clinical presentations include angina/acute coronary syndrome, myocardial infarction, thrombosis, embolization, and aneurysm presenting as a cardiac tamponade [2]. Coronary angiography is the most common imaging modality used for the evaluation of CAAs [2]. It is important to note that, in the setting of giant CAAs, when undergoing coronary angiography, a forceful and prolonged injection of contrast is necessary to avoid misinterpreting slow aneurysmal filling as in situ thrombosis [2].CT is helpful in patients with giant CAAs to provide a more precise assessment of potential mechanical complications [2].

## Case presentation

**Figure 1:**
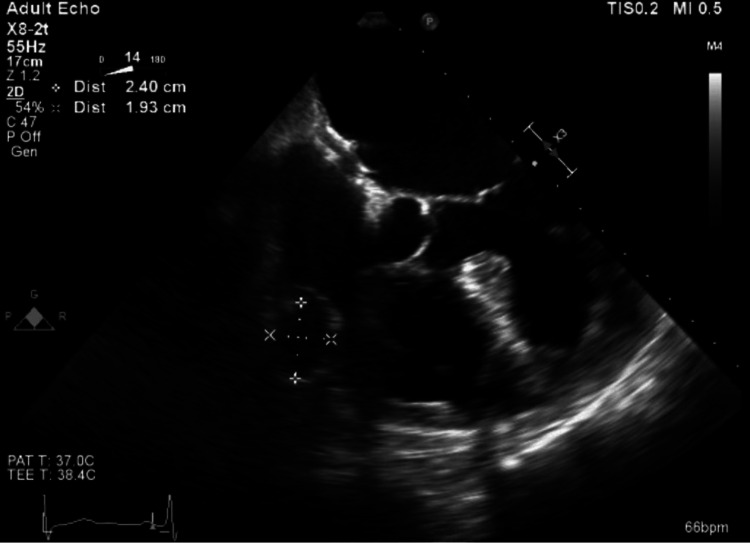
Echocardiogram showing a giant right coronary artery aneurysm

An 82-year-old man presented to the hospital complaining of dark tarry stools, worsening fatigue, generalized weakness, arm numbness, and dizziness for the past two days. His past medical history was significant for paroxysmal atrial fibrillation, colon cancer status post resection, noninsulin-dependent diabetes mellitus, stage 3 chronic kidney disease (CKD), right iliac aneurysm status post endovascular repair, and abdominal aortic aneurysm status post repair. Further evaluation revealed he had an episode of acute gastrointestinal bleeding and a multi-infarct stroke. During his hospitalization, a carotid Doppler and transthoracic echocardiogram were performed to determine a possible underlying cardiovascular etiology for a thromboembolic event. The transthoracic echocardiogram revealed a ring-like structure in the right atrium adjacent to the tricuspid valve. He presented to the office post-hospitalization and a transesophageal echocardiogram (TEE) was recommended and performed, which revealed an echo-free ring-like lesion in the right atrium on the lateral aspect, near the tricuspid annulus (Figure 1).

**Figure 2:**
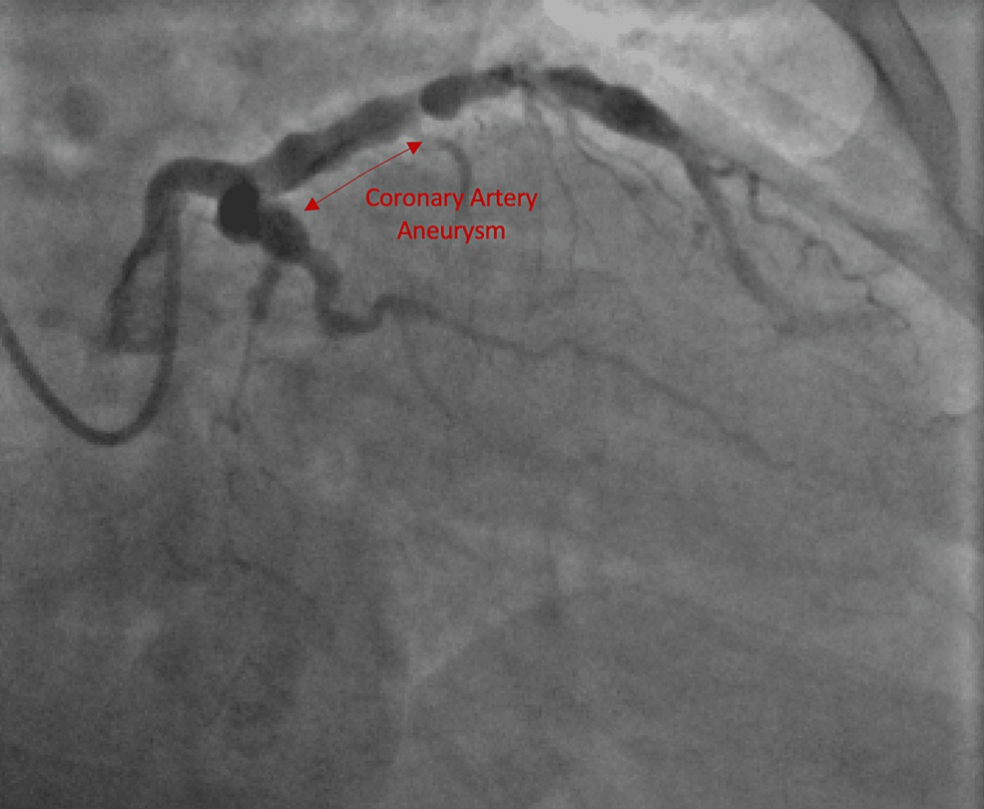
Cardiac catheterization demonstrating aneurysmal left anterior descending artery and left circumflex artery

There was no evidence of intracardiac thrombi or vegetation and no blood flow was visualized to the structure. The mass was initially believed to most likely be a right atrial cyst although the etiology was not clear. No evidence of a cardiac etiology for a thromboembolic event could be identified at this point. Coronary arteriography with left heart catheterization, left ventriculography, and right iliac arteriography were performed. Left ventriculography revealed normal left ventricular (LV) function with an estimated ejection fraction of 60%. Coronary arteriography revealed aneurysms of the coronary arteries. The left main coronary artery was patent. Coronary arteriography showed that the left anterior descending artery (LAD) was aneurysmal throughout (Figure 2) and that the middle portion of the LAD had 75% stenosis and total occlusion of the distal LAD.

**Figure 3:**
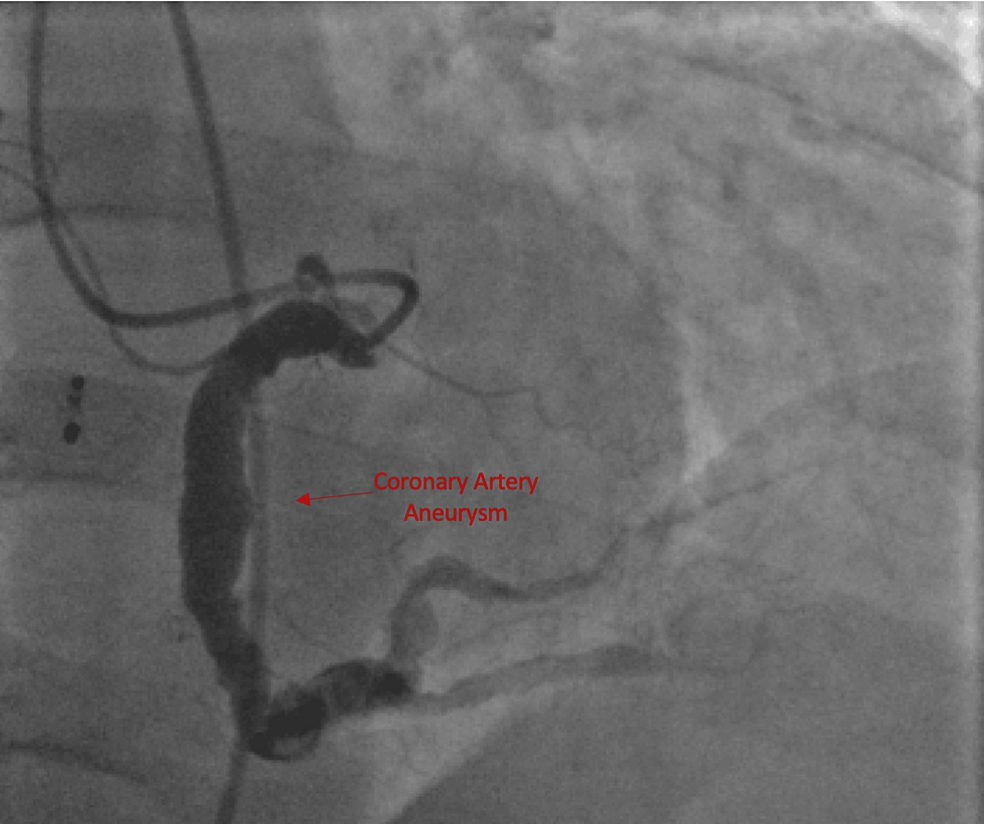
Cardiac catheterization demonstrating aneurysmal right coronary artery

There was a severe disease of the diagonals. The left circumflex artery was aneurysmal with no flow-limiting lesion noted (Figure 2). There was an extremely large aneurysm located in the midportion of the right coronary artery, approximately 5 cm of the right coronary artery, with no signs of thrombus (Figure 3).

No flow-limiting lesions were noted. His right iliac endovascular graft appeared patent. Loop recorder implantation was performed for recurrent cerebrovascular accidents (CVAs), cryptogenic stroke, and a history of atrial fibrillation. The treatment options for the aneurysm as well as the risks and benefits of each approach were discussed with him. He opted for regular follow-ups of his aneurysm given that his multiple comorbidities made him a high-risk patient for surgical intervention.

## Discussion

CAAs are dangerous cardiovascular conditions that require close care to prevent morbidity and mortality. Etiologies for their development include atherosclerosis, congenital causes, connective tissue disease, dynamic wall stress, and infectious etiologies [2-3]. They have also been reported as rare events following percutaneous interventions including placement of drug-eluting stents (DESs) [3-4]. A retrospective study of 1197 patients who underwent stenting found 15 CAAs (1.25 percent) during follow-up angiography [4]. The mean time between placement and CAA diagnosis was 313 days +/- 194 days [4]. We have insufficient evidence to conclude any inflammatory or direct wall injury to be the etiology for his giant CAA. Furthermore, he had no past medical history relevant to suggest congenital defects such as connective tissue disorders or vasculitis such as Kawasaki Disease. The etiology of his giant CAA remains to be confirmed at this time.

Detection of giant CAAs is usually through cardiac catheterization [2]. Nevertheless, catheterization can lead to the formation of aneurysms. We found our patient's aneurysm through noninvasive imaging. One case in the literature was similar to ours and details the detection of a giant CAA through echocardiography [5]. Echocardiography may be a novel way to detect CAAs [5].^ ^

The management of CAAs proves challenging given the lack of randomized control trials and large-scale data. Other factors that increase the challenge of proper management include unconfirmed etiologies of CAAs. Additionally, in patients presenting with angina or myocardial infarction (MI) of the suspected aneurysmal culprit, percutaneous and surgical interventions are associated with technical challenges. Most of the current treatment options are based on small case series and anecdotical evidence. Treatment options include medical management, percutaneous coronary intervention, and surgical intervention [2].

Medical management is recommended in older patients who have an isolated CAA with suspected atherosclerotic etiology. In this subset of patients, an aggressive risk factor modification is warranted [2].​​​​​​​Anticoagulation and antiplatelet therapy remain controversial in the setting of CAA given the lack of available large-scale data. Avoidance of nitrates is suggested as they have been shown to exacerbate ischemia in patients with large, isolated CAAs [2].

Data regarding percutaneous coronary intervention is limited. It is even more limited in the setting of a patient presenting with a giant coronary artery aneurysm outside of the setting of acute coronary syndrome (ACS). In the setting of ACS, the goal is to restore flow; however, patients in this group have higher mortality and stent thrombosis [2]. Outside the scope of our case, a covered stent exclusion for saccular and small pseudoaneurysms not involving a major side branch is recommended [2]. In the setting of major side branch involvement, balloon or stent-assisted coil embolization is recommended. In the setting of a giant CAA, left main involvement, or in the setting of multiple CAAs, surgical resection is the preferred method of choice [2]. Amplatzer occlusion or coil embolization with or without percutaneous coronary intervention may be a reasonable alternative to surgical intervention in patients with saphenous vein graft aneurysms [2].

## Conclusions

This 82-year-old man was found to have a giant right CAA and multivessel aneurysms during comprehensive evaluation for his multi-infarct stroke. The use of echocardiography ultimately led to the discovery of his CAA; echocardiography as a form of imaging modality for the detection of a CAA was unique to this case. Traditionally cardiac catheterization has been the diagnostic imaging modality; however, catheterization can also lead to the formation of aneurysms. Echocardiography may be a safer modality for the initial detection of coronary aneurysms. Our patient ultimately decided to pursue close follow-up and declined surgical or percutaneous intervention. He was stable at his six-month follow-up.
